# Machine learning integration identifying an eight-gene diagnostic signature for acute mountain sickness

**DOI:** 10.3389/fmed.2025.1688025

**Published:** 2025-11-18

**Authors:** Dan Yang, Xinyao Yin, Qian Li, Xin Wang, Junqiang Gou, Mengmeng Liu, Xinman Peng, Zhuxing Xu, Xiao Yang, Wenyan Jia, Haiwen Tang, Qiuli Zhang, Feng Yang, Xiaofeng Wang, Rui Wang

**Affiliations:** 1 General Hospital of Xinjiang Military Command, Urumqi, China; 2 Xinjiang Medical University, Urumqi, China; 3 New York University Shanghai, Shanghai, China; 4 The Nineth Medical Center of PLA General Hospital Gynaecology and Obstetrics, Beijing, China; 5 Center for Disease Control and Prevention of Ministry Security in Xinjiang Military Region, Urumqi, China

**Keywords:** acute mountain sickness, machine learning, diagnostic signature, single-cell RNA-seq, personalized medicine

## Abstract

**Background:**

Acute mountain sickness (AMS) is highly prevalent at high altitudes, with estimated incidence rates ranging from 25 to 90%. However, current AMS diagnosis primarily relies on self-reported questionnaires, highlighting the need for reliable biomarkers. Thus, we aimed to establish a diagnostic model for AMS.

**Methods:**

We applied scRNA-seq (*n* = 10) and bulk RNA-seq (*n* = 192) to identify AMS-associated genes. Then, we constructed AMS diagnostic model by machine learning. We also assessed the expression levels of AMS-related gene signatures using Quantitative PCR. Finally, we explored the mechanism of AMS-associated signatures by epigenetic analyses and KEGG pathway enrichment.

**Results:**

We analyzed cellular heterogeneity through scRNA-seq data, revealing significant enrichment of myeloid (MD) and platelet (PLT) cells during AMS progression. Subsequently, we identified 526 differentially expressed genes (DEGs) associated with the progression of AMS using pseudobulk differential expression analysis on the MD and PLT subsets between the AMS and control groups. We further screened for AMS-associated genes using bulk RNA-seq based differential analysis and WGNCA. Finally, we screened 12 AMS-related genes using scRNA-seq and bulk-RNA-seq data. These genes were utilized as features across 113 distinct combinations of machine learning models to develop an AMS diagnostic model. The model of Stepglm[both] + NaiveBayes (ATP6V0C, BCL2A1, CD52, CSTA, GZMA, HINT1, PFDN5, and RNF11) demonstrated optimal diagnostic accuracy. It obtained an AUC of 0.948 on the training cohort (*n* = 160) and maintained robust performance on external validation cohorts, with AUCs of 0.818 (GSE103940 = 22) and 0.760 (GSE75665 = 10). Using qPCR, we confirmed that the mRNA levels of the model genes were aligned with the transcriptome data (*p* < 0.05). Based on the epigenetic analyses, we found the AMS signatures might regulate by the histone and m6A methylation. Furthermore, pathway analysis revealed significant enrichment of these signature genes in immune-related signaling pathways and oxidative stress (adjusted *p* < 0.05).

**Conclusion:**

Using machine learning, we identified and validated a minimal blood biomarker signature for AMS diagnosis. This approach offered a practical approach for the early detection of AMS, especially in resource-limited populations residing in high-altitude regions.

## Introduction

Annually, over 40 million individuals visit high-altitude areas (>2,500 m), and approximately 140 million people permanently reside in such regions (1). This accessibility is largely due to the expansion of modern transportation infrastructure. Acute mountain sickness (AMS) is the most prevalent altitude-related condition, with estimated incidence rates ranging from 25 to 90% (2, 3). However, the diagnosis of AMS primarily relies on subjective scoring systems, which can result in misdiagnosis and delayed treatment (4). Therefore, there is an urgent need to develop an objective diagnostic method to enhance the accuracy and timeliness of AMS diagnosis.

The diagnosis of AMS currently relies on the subjective symptom scores from the internationally recognized Lake Louise Scoring (LLS) system (5). However, dependence on subjective symptoms makes diagnosis susceptible to interference from multiple factors. Although researches have explored using objective indicators (6, 7) (e.g., physiological, biochemical, psychological, genetic, altitude, and geographic factors) to support AMS diagnosis, these methods generally require professional equipment and the participation of experienced physicians, resulting in implementation difficulties in high-altitude environments. Recent advances demonstrated that applying high-throughput sequencing data significantly enhanced precision oncology (8, 9). Notably, integrated multi-omics analyses developed robust prognostic signatures across malignancies (e.g., glioma, pancreatic cancer) (10, 11). Ensemble machine learning frameworks outperform conventional indicators and biomarkers (mean C-index > 0.7; AUC > 0.78), identified clinically actionable signatures to diagnose diseases. Together, previous studies established a methodological basis for developing an accurate AMS diagnostic model through the combined application of omics data and machine learning techniques.

In this study, we elucidated critical pathogenic mediators driving AMS progression, through systematic integration of scRNA-seq and bulk RNA-seq. Subsequently, we constructed a robust diagnostic signature for AMS using the machine learning methods. Finally, we applied two independent cohort datasets to validate the AMS signature. This study provided a practical model for AMS diagnoses in the resource-limited high-altitude regions.

## Materials and methods

### Sample collection

All subjects of training cohort transported to Thirty-li Barracks Medical Station (altitude of 3,700 m) from Chengdu (altitude of 500 m) via air and ground transportation (the total journey lasted 2 days). Acute Mountain Sickness (AMS) was assessed 6 h after passive ascent to an altitude of 3,700 m according to the 2018 Lake Louise Scoring System (LLS), with AMS defined as headache accompanied by a total LLS score ≥3. To perform scRNA-seq and bulk RNA-seq, we isolated peripheral blood mononuclear cells (PBMCs) from patients with AMS and healthy volunteers. Detailed clinical characteristics of all participants were summarized in Table 1. We performed scRNA-seq on five AMS and five healthy PBMC samples. Bulk RNA-seq was also conducted on corresponding samples from 80 AMS patients and 80 healthy controls. Furthermore, 32 Bulk RNA-seq samples from the GSE103940 and GSE6565 datasets were downloaded to validate the AMS diagnostic model.

**Table 1:** The clinical characteristics of samples.

Group	LLS	Age (year)	BMI (kg/m^2^)	SpO2(%)	Altitude (m)	Timing	Timing of blood draws
AMS1	4	25	23.7	98	3,700	2 days	Within 24 h of diagnosis
AMS2	5	26	21.3	97	3,700	2 days	Within 24 h of diagnosis
AMS3	3	28	23.4	97	3,700	2 days	Within 24 h of diagnosis
AMS4	5	25	21	95	3,700	2 days	Within 24 h of diagnosis
AMS5	9	25	20.5	97	3,700	2 days	Within 24 h of diagnosis
AMS6	7	24	19.9	98	3,700	2 days	Within 24 h of diagnosis
AMS7	6	27	21.3	97	3,700	2 days	Within 24 h of diagnosis
AMS8	3	25	22.6	97	3,700	2 days	Within 24 h of diagnosis
AMS9	3	25	22.7	95	3,700	2 days	Within 24 h of diagnosis
AMS10	4	25	20.9	96	3,700	2 days	Within 24 h of diagnosis
AMS11	5	26	24.9	96	3,700	2 days	Within 24 h of diagnosis
AMS12	5	29	22.7	98	3,700	2 days	Within 24 h of diagnosis
AMS13	4	26	24.4	97	3,700	2 days	Within 24 h of diagnosis
AMS14	4	25	22.8	96	3,700	2 days	Within 24 h of diagnosis
AMS15	8	27	20.1	99	3,700	2 days	Within 24 h of diagnosis
AMS16	3	26	21.2	97	3,700	2 days	Within 24 h of diagnosis
AMS17	6	26	23.2	99	3,700	2 days	Within 24 h of diagnosis
AMS18	6	26	20.4	97	3,700	2 days	Within 24 h of diagnosis
AMS19	5	25	23.9	99	3,700	2 days	Within 24 h of diagnosis
AMS20	3	27	21.6	96	3,700	2 days	Within 24 h of diagnosis
AMS21	5	26	22.7	97	3,700	2 days	Within 24 h of diagnosis
AMS22	3	27	20.6	98	3,700	2 days	Within 24 h of diagnosis
AMS23	4	25	23.9	99	3,700	2 days	Within 24 h of diagnosis
AMS24	5	26	21.7	97	3,700	2 days	Within 24 h of diagnosis
AMS25	5	27	21.8	96	3,700	2 days	Within 24 h of diagnosis
AMS26	3	27	21.3	97	3,700	2 days	Within 24 h of diagnosis
AMS27	5	27	20.2	95	3,700	2 days	Within 24 h of diagnosis
AMS28	3	24	21	97	3,700	2 days	Within 24 h of diagnosis
AMS29	6	28	21.3	96	3,700	2 days	Within 24 h of diagnosis
AMS30	3	25	23.8	98	3,700	2 days	Within 24 h of diagnosis
AMS31	6	26	23.1	97	3,700	2 days	Within 24 h of diagnosis
AMS32	3	27	20.3	96	3,700	2 days	Within 24 h of diagnosis
AMS33	3	26	22.4	97	3,700	2 days	Within 24 h of diagnosis
AMS34	5	26	21.5	95	3,700	2 days	Within 24 h of diagnosis
AMS35	6	25	19	96	3,700	2 days	Within 24 h of diagnosis
AMS36	3	24	21	99	3,700	2 days	Within 24 h of diagnosis
AMS37	3	23	22.5	98	3,700	2 days	Within 24 h of diagnosis
AMS38	3	25	21.8	95	3,700	2 days	Within 24 h of diagnosis
AMS39	5	28	20.7	96	3,700	2 days	Within 24 h of diagnosis
AMS40	5	26	23.4	95	3,700	2 days	Within 24 h of diagnosis
AMS41	5	27	21.2	95	3,700	2 days	Within 24 h of diagnosis
AMS42	3	26	22	97	3,700	2 days	Within 24 h of diagnosis
AMS43	3	28	21.5	96	3,700	2 days	Within 24 h of diagnosis
AMS44	6	26	23.4	95	3,700	2 days	Within 24 h of diagnosis
AMS45	5	28	20.9	98	3,700	2 days	Within 24 h of diagnosis
AMS46	4	27	21.8	97	3,700	2 days	Within 24 h of diagnosis
AMS47	3	25	25.8	99	3,700	2 days	Within 24 h of diagnosis
AMS48	5	27	22.7	95	3,700	2 days	Within 24 h of diagnosis
AMS49	4	26	21.7	99	3,700	2 days	Within 24 h of diagnosis
AMS50	6	28	23.3	96	3,700	2 days	Within 24 h of diagnosis
AMS51	6	24	22.2	96	3,700	2 days	Within 24 h of diagnosis
AMS52	5	26	20.4	99	3,700	2 days	Within 24 h of diagnosis
AMS53	5	25	21.6	96	3,700	2 days	Within 24 h of diagnosis
AMS54	3	27	20.7	95	3,700	2 days	Within 24 h of diagnosis
AMS55	3	25	23	95	3,700	2 days	Within 24 h of diagnosis
AMS56	3	29	19.8	96	3,700	2 days	Within 24 h of diagnosis
AMS57	4	26	21.4	99	3,700	2 days	Within 24 h of diagnosis
AMS58	4	27	22.2	96	3,700	2 days	Within 24 h of diagnosis
AMS59	3	27	21.4	96	3,700	2 days	Within 24 h of diagnosis
AMS60	3	26	23.7	97	3,700	2 days	Within 24 h of diagnosis
AMS61	3	27	21.5	96	3,700	2 days	Within 24 h of diagnosis
AMS62	4	27	23.1	95	3,700	2 days	Within 24 h of diagnosis
AMS63	3	24	25.3	97	3,700	2 days	Within 24 h of diagnosis
AMS64	4	27	20.8	96	3,700	2 days	Within 24 h of diagnosis
AMS65	3	27	19.8	95	3,700	2 days	Within 24 h of diagnosis
AMS66	4	24	23.3	97	3,700	2 days	Within 24 h of diagnosis
AMS67	5	26	21.5	98	3,700	2 days	Within 24 h of diagnosis
AMS68	4	27	22.6	97	3,700	2 days	Within 24 h of diagnosis
AMS69	3	27	21.2	99	3,700	2 days	Within 24 h of diagnosis
AMS70	3	26	19.7	99	3,700	2 days	Within 24 h of diagnosis
AMS71	4	27	24	99	3,700	2 days	Within 24 h of diagnosis
AMS72	3	25	23.8	99	3,700	2 days	Within 24 h of diagnosis
AMS73	3	26	19.3	97	3,700	2 days	Within 24 h of diagnosis
AMS74	6	26	19.2	99	3,700	2 days	Within 24 h of diagnosis
AMS75	8	28	20.9	98	3,700	2 days	Within 24 h of diagnosis
AMS76	4	26	19.8	98	3,700	2 days	Within 24 h of diagnosis
AMS77	6	26	23.8	95	3,700	2 days	Within 24 h of diagnosis
AMS78	3	25	25.2	99	3,700	2 days	Within 24 h of diagnosis
AMS79	7	26	23.2	96	3,700	2 days	Within 24 h of diagnosis
AMS80	5	26	21.3	96	3,700	2 days	Within 24 h of diagnosis
Control1	0	26	20.9	95	3,700	2 days	Within 24 h of diagnosis
Control2	0	24	20.3	96	3,700	2 days	Within 24 h of diagnosis
Control3	0	28	20.1	97	3,700	2 days	Within 24 h of diagnosis
Control4	0	24	21.1	99	3,700	2 days	Within 24 h of diagnosis
Control5	1	26	23.3	95	3,700	2 days	Within 24 h of diagnosis
Control6	0	26	21.9	95	3,700	2 days	Within 24 h of diagnosis
Control7	0	25	23.2	98	3,700	2 days	Within 24 h of diagnosis
Control8	0	23	20.5	95	3,700	2 days	Within 24 h of diagnosis
Control9	0	25	21.1	98	3,700	2 days	Within 24 h of diagnosis
Control10	0	25	21.5	95	3,700	2 days	Within 24 h of diagnosis
Control11	0	26	22.4	96	3,700	2 days	Within 24 h of diagnosis
Control12	0	24	17.5	98	3,700	2 days	Within 24 h of diagnosis
Control13	1	27	22.6	98	3,700	2 days	Within 24 h of diagnosis
Control14	0	25	21.9	99	3,700	2 days	Within 24 h of diagnosis
Control15	0	25	21.2	96	3,700	2 days	Within 24 h of diagnosis
Control16	0	26	22.2	96	3,700	2 days	Within 24 h of diagnosis
Control17	0	26	19.5	95	3,700	2 days	Within 24 h of diagnosis
Control18	0	25	21.5	96	3,700	2 days	Within 24 h of diagnosis
Control19	1	25	21.2	97	3,700	2 days	Within 24 h of diagnosis
Control20	1	23	21.5	98	3,700	2 days	Within 24 h of diagnosis
Control21	2	24	20.3	97	3,700	2 days	Within 24 h of diagnosis
Control22	0	24	22.1	95	3,700	2 days	Within 24 h of diagnosis
Control23	0	24	23	99	3,700	2 days	Within 24 h of diagnosis
Control24	0	25	21.5	95	3,700	2 days	Within 24 h of diagnosis
Control25	1	25	20.4	96	3,700	2 days	Within 24 h of diagnosis
Control26	0	24	18.6	96	3,700	2 days	Within 24 h of diagnosis
Control27	0	26	20	95	3,700	2 days	Within 24 h of diagnosis
Control28	1	25	21.4	95	3,700	2 days	Within 24 h of diagnosis
Control29	0	24	21	98	3,700	2 days	Within 24 h of diagnosis
Control30	0	26	22.2	97	3,700	2 days	Within 24 h of diagnosis
Control31	0	24	20.3	95	3,700	2 days	Within 24 h of diagnosis
Control32	0	25	19.8	97	3,700	2 days	Within 24 h of diagnosis
Control33	0	25	21.5	97	3,700	2 days	Within 24 h of diagnosis
Control34	0	24	22.3	99	3,700	2 days	Within 24 h of diagnosis
Control35	1	25	21	99	3,700	2 days	Within 24 h of diagnosis
Control36	0	25	21.4	95	3,700	2 days	Within 24 h of diagnosis
Control37	0	26	21.1	99	3,700	2 days	Within 24 h of diagnosis
Control38	1	25	23.2	98	3,700	2 days	Within 24 h of diagnosis
Control39	0	26	20.4	97	3,700	2 days	Within 24 h of diagnosis
Control40	1	25	21.3	98	3,700	2 days	Within 24 h of diagnosis
Control41	0	24	22.2	95	3,700	2 days	Within 24 h of diagnosis
Control42	0	25	20.2	95	3,700	2 days	Within 24 h of diagnosis
Control43	0	25	20.6	96	3,700	2 days	Within 24 h of diagnosis
Control44	0	26	17.7	99	3,700	2 days	Within 24 h of diagnosis
Control45	0	25	20.8	95	3,700	2 days	Within 24 h of diagnosis
Control46	0	25	21.2	97	3,700	2 days	Within 24 h of diagnosis
Control47	0	24	20	98	3,700	2 days	Within 24 h of diagnosis
Control48	0	26	20.7	97	3,700	2 days	Within 24 h of diagnosis
Control49	2	24	20.4	97	3,700	2 days	Within 24 h of diagnosis
Control50	0	24	22.1	98	3,700	2 days	Within 24 h of diagnosis
Control51	1	26	20.8	98	3,700	2 days	Within 24 h of diagnosis
Control52	2	25	18.5	99	3,700	2 days	Within 24 h of diagnosis
Control53	0	26	21.6	97	3,700	2 days	Within 24 h of diagnosis
Control54	2	26	20.7	97	3,700	2 days	Within 24 h of diagnosis
Control55	0	26	20.4	96	3,700	2 days	Within 24 h of diagnosis
Control56	2	25	20.8	95	3,700	2 days	Within 24 h of diagnosis
Control57	1	26	20.2	97	3,700	2 days	Within 24 h of diagnosis
Control58	1	26	20.6	97	3,700	2 days	Within 24 h of diagnosis
Control59	0	25	21.4	99	3,700	2 days	Within 24 h of diagnosis
Control60	1	23	20	97	3,700	2 days	Within 24 h of diagnosis
Control61	1	27	19.8	96	3,700	2 days	Within 24 h of diagnosis
Control62	1	26	21.7	95	3,700	2 days	Within 24 h of diagnosis
Control63	0	26	19.8	95	3,700	2 days	Within 24 h of diagnosis
Control64	1	26	20.8	96	3,700	2 days	Within 24 h of diagnosis
Control65	0	24	20.8	95	3,700	2 days	Within 24 h of diagnosis
Control66	1	27	22.1	97	3,700	2 days	Within 24 h of diagnosis
Control67	0	26	23.4	99	3,700	2 days	Within 24 h of diagnosis
Control68	0	26	22.1	98	3,700	2 days	Within 24 h of diagnosis
Control69	1	25	21	95	3,700	2 days	Within 24 h of diagnosis
Control70	1	22	23.2	96	3,700	2 days	Within 24 h of diagnosis
Control71	1	26	20.4	96	3,700	2 days	Within 24 h of diagnosis
Control72	1	25	21.2	95	3,700	2 days	Within 24 h of diagnosis
Control73	1	25	21	95	3,700	2 days	Within 24 h of diagnosis
Control74	0	26	18.9	96	3,700	2 days	Within 24 h of diagnosis
Control75	0	26	21	95	3,700	2 days	Within 24 h of diagnosis
Control76	1	26	21	96	3,700	2 days	Within 24 h of diagnosis
Control77	0	27	20.8	98	3,700	2 days	Within 24 h of diagnosis
Control78	0	25	23.5	99	3,700	2 days	Within 24 h of diagnosis
Control79	0	26	22.7	96	3,700	2 days	Within 24 h of diagnosis
Control80	0	24	19.4	96	3,700	2 days	Within 24 h of diagnosis

### Analysis of single-cell transcriptome profiles

The PBMC samples were processed using established protocols (12, 13). The single cells (>90% viability) were isolated on the 10x Chromium platform (10x Genomics) to generate raw data. The count matrix of features was generated by CellRanger (v8.0.0), standardized using the SCTransform method (v0.3.5), and batch-integrated via Harmony (v0.1.1) (13). The following quality control criteria were applied using Seurat (v4.0.2): cells with >500 detected genes, <4,000 detected genes, and <10% mitochondrial gene content (14). Canonical markers were used to annotate major cell types (15). Finally, pseudobulk differential expression analysis employed thresholds of |log2(fold change)| > 0.2 and adjusted *p* < 0.05 to identify the DEGs (16).

### Identification of AMS-associated genes using bulk RNA-seq

We performed bulk RNA-seq on 96 AMS patients and 96 healthy controls to identify genes related to AMS. First, sequencing libraries were prepared using high-quality RNA (RIN > 7.0) and sequenced on the Illumina platform. Next, the count matrix was obtained by STAR software (v2.7.2a) (17) and htseq-count (v2.05) (18). Finally, DEGs were identified by DESeq2 (v1.40.2) (19) with the cutoff of adjusted *p* < 0.05 and |log2(fold change)| > 0.5 (20, 21).

We also identified genes associated with AMS using WGCNA (20). Modules were constructed using topological overlap matrix (TOM)-based dissimilarity with dynamic tree cutting, applying the following parameters: *β* = 16, minModuleSize = 50, mergeCutHeight = 0.15, and deepSplit = 2. The module most significantly correlated with AMS was then identified based on the highest absolute correlation coefficient and *p* < 0.05 (20). The genes in this module were designated as putative AMS-associated genes. Finally, genes shared across scRNA-seq data, differentially expressed genes, and WGCNA modules were selected as candidate genes related with AMS.

### Identification of the AMS diagnostic signature using machine learning

We developed 113 diagnostic models for AMS based on combinations of 10 machine-learning algorithms based on the previous studies (8, 9). Subsequently, model performance was evaluated on two independent datasets, GSE103940 and GSE75665, employing the concordance index (C-index), confusion matrices, Brier scores, and the Hosmer-Lemeshow test.

### Validation of AMS-associated signatures

We evaluated the expression levels of model genes using quantitative real-time PCR (qPCR) in six AMS and six control samples, with each sample run in six technical replicates (21). Samples were blinded during RNA processing and QPCR setup. The primers were summarized in Supplementary Table S1. qPCR amplification was performed under standardized cycling conditions: initial denaturation at 95 °C for 3 min, followed by 40 cycles of denaturation at 95 °C for 10 s and annealing/extension at 60 °C for 30 s. For normalization, the GAPDH served as the endogenous control. Finally, relative quantification was performed using the comparative threshold cycle (2^−ΔΔCt^) method.

### Identifying the mechanism of AMS-associated signatures

To identify the upstream mechanisms of the AMS-associated signature, we defined the AMS score as the average expression of eight signature genes (ATP6V0C, BCL2A1, CD52, CSTA, GZMA, HINT1, PFDN5, RNF11). Subsequently, we identified potential regulators associated with this signature by calculating pairwise Spearman correlation based on the AMS score. We then explored the downstream mechanisms of the AMS signature using KEGG pathway enrichment with the cutoff of adjusted *p* < 0.05.

### Statistical analysis

We performed statistical analyses by R (v4.3.3). First, data normality was assessed via the Shapiro–Wilk test and variance homogeneity evaluated using Levene’s test prior to parametric testing. Subsequently, unpaired two-tailed Student’s t-tests were applied to comparisons satisfying these assumptions. Statistical significance was defined as *p* value < 0.05.

## Results

### Identification of AMS related genes through scRNA-seq

**Figure 1:**
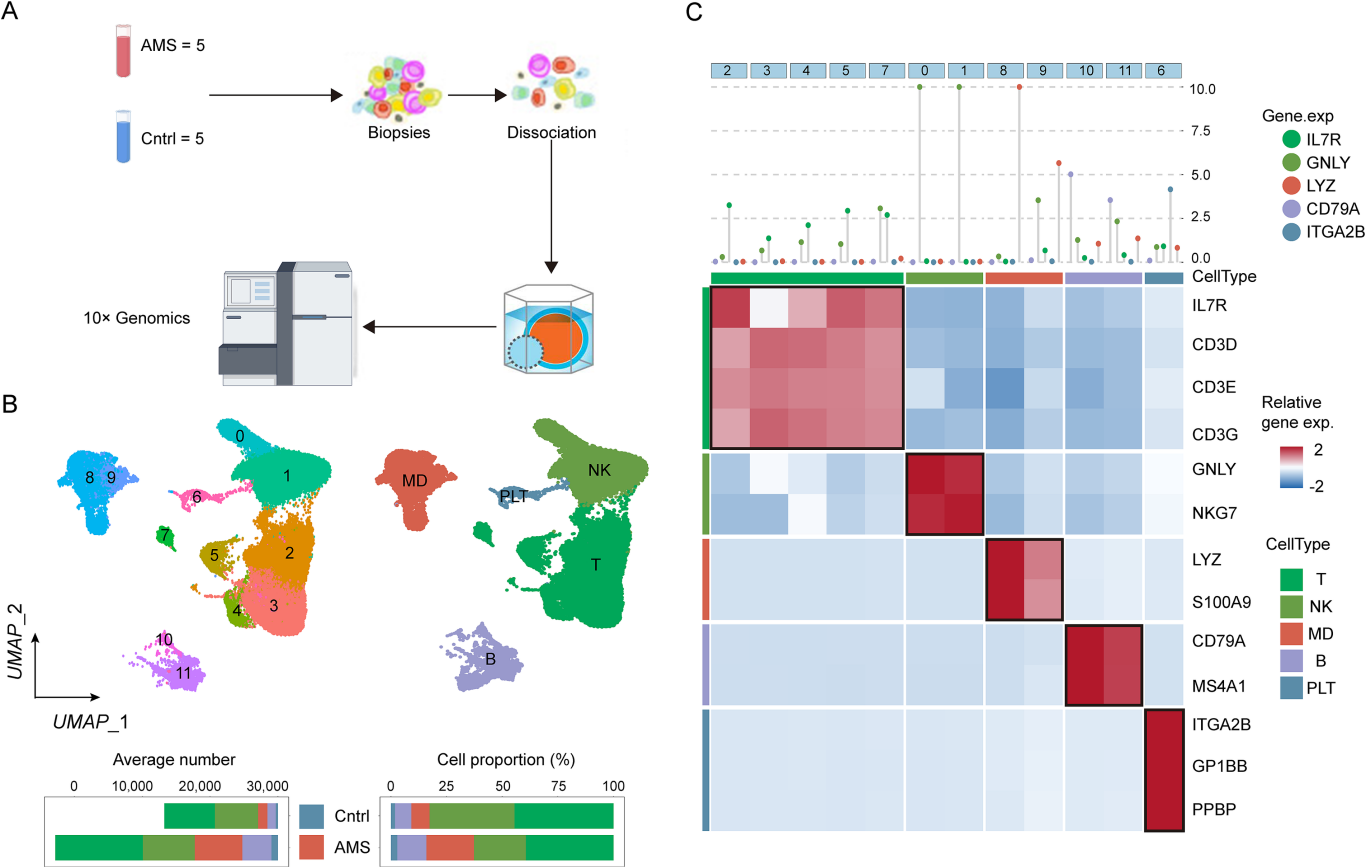
Identifying the heterogeneity of AMS microenvironment by scRNA-seq. **(A)** Schematic workflow illustrated the procedures of scRNA-seq. **(B)** The UMAP showed cluster identity (left) and major cell types (right) for 26,169 cells obtained from 10 specimens (5 Control and 5 AMS). **(C)** These results were depicted in a two-layered heatmap highlighting selected canonical markers for each cell type. The upper layer presented the mean expression of these markers, whereas the lower layer displayed a relative expression map for the corresponding marker genes. The relative expression values were scaled via mean centering and transformed to a range of −2 to 2.

We generated a single-cell transcriptomic atlas consisting of 26,169 single cells derived from five AMS patients and five healthy controls (Figure 1A). Subsequently, we applied unsupervised clustering to identify 12 distinct clusters (Figure 1B). These clusters were then annotated into five major cell types (Figure 1B) using canonical markers (Figure 1C). Based on cell proportion analysis, we found that myeloid-derived (MD) cells and platelet (PLT) cells were increased in AMS patients compared to healthy controls. This increase indicated that MD and PLT cells played a critical role in the progression of AMS. Consequently, to identify the potential candidates associated with AMS, we screened for differentially expressed genes (DEGs) in MD (Supplementary Table S2) and PLT cells (Supplementary Table S3) between the AMS and control groups using pseudobulk differential expression analysis.

### Identification of AMS-associated genes by differential gene expression analysis of bulk RNA-Seq data

**Figure 2:**
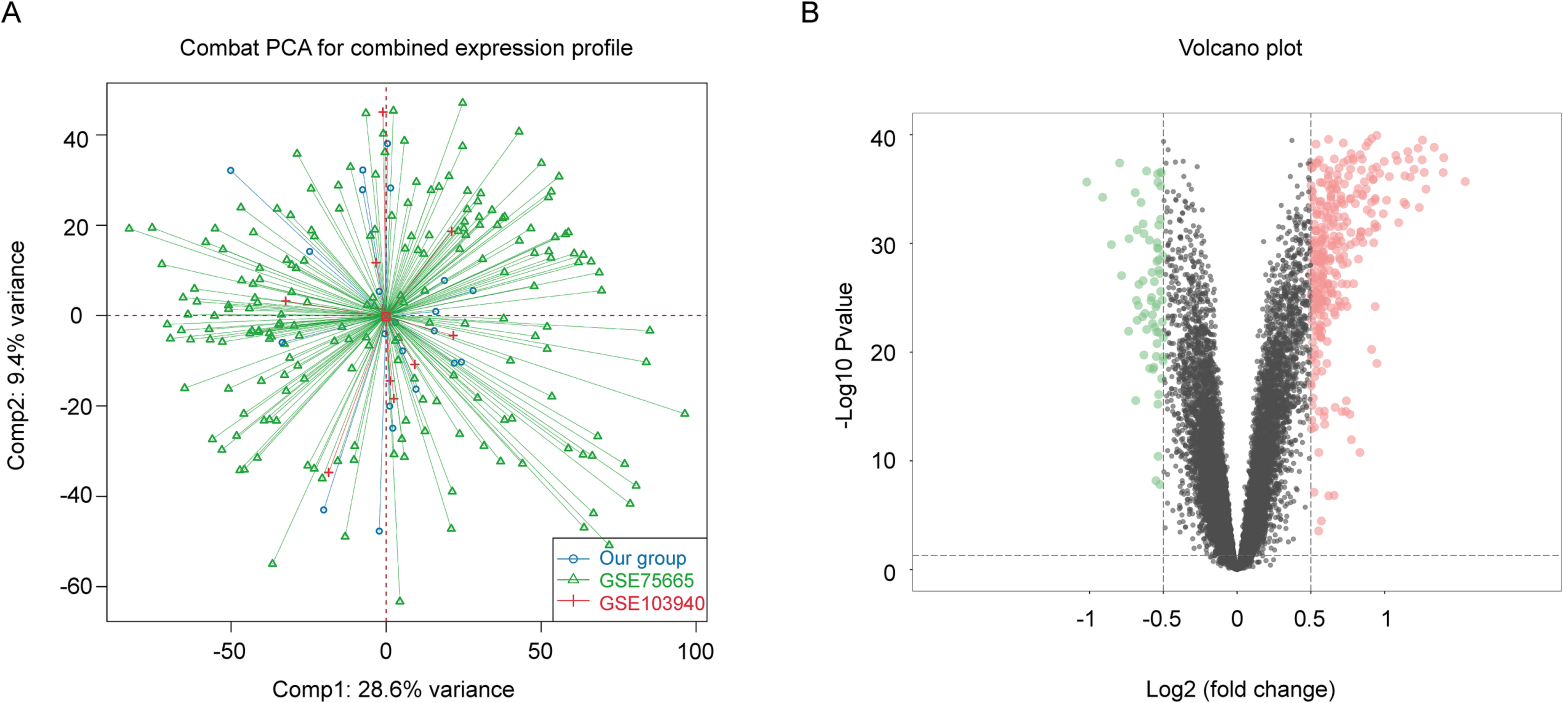
Identification of AMS-Associated genes by differential gene expression analysis of bulk RNA-Seq Data. **(A)** We reduced non-biological technical batch effects using the ComBat method. **(B)** The DEGs between the AMS and control groups (AMS = 96 and the control = 96) were visualized as a volcano plot. Horizontal and vertical gray dotted lines indicated the threshold of |log2(fold change)| > 0.5 and adjusted *p* < 0.05. The Red (green) dots indicated significantly upregulated (downregulated) genes. DEGs: the differentially expressed genes.

To improve the accuracy of identifying AMS-associated genes, we first integrated our in-house AMS bulk RNA-seq dataset with data from public repositories using the ComBat algorithm, resulting in a consolidated dataset comprising 96 AMS samples and 96 controls (Figure 2A). Subsequently, we performed differential expression analysis on this integrated dataset to screen for AMS-related gene sets. By applying predefined thresholds for DEGs (|log2 (fold change)| > 0.5 and adjusted *p* < 0.05), we identified 419 significantly differentially expressed genes (Figure 2B; Supplementary Table S4). Additionally, using the ssizeRNA package (v1.3.3) (22), we estimated a minimum requirement of 69 samples to achieve 80% statistical power, given the specified parameters (proportion of non-differentially expressed genes *π*₀ = 0.98; fold change thresholds fold change = 1.4 or 1.5). The actual cohort size of 96 exceeds this minimum, ensuring that the sampling design provides sufficient power for robust detection of target DEGs with reliable false discovery rate control.

### Identification of AMS-associated genes by WGCNA based on bulk RNA-Seq

**Figure 3:**
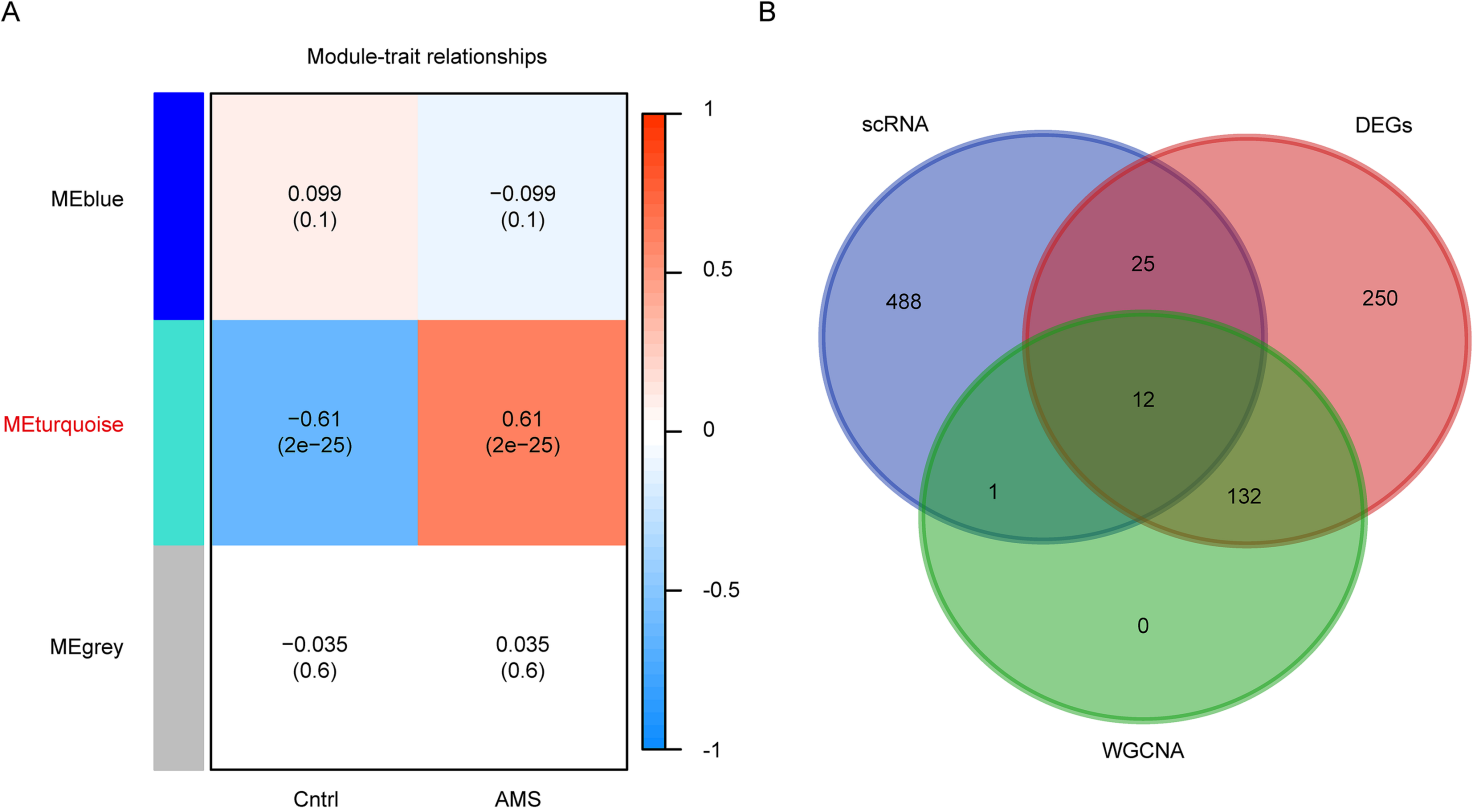
Identification of AMS-Associated genes by WGCNA of bulk RNA-Seq Data. **(A)** The correlation of each module with the clinical trait was visualized as a heatmap. The correlation coefficient and the *p* values are included in each cell. Each module was labeled with different colors. **(B)** Overlapping genes were identified by multi-omics approaches.

To further screen AMS-related genes, we performed WGCNA based on bulk RNA-seq data. This analysis identified three gene co-expression modules based on module-trait relationships (Figure 3A). The turquoise module exhibited a strong association with the AMS group, using a cutoff of the highest absolute correlation coefficient and *p* < 0.05 (Figure 3A). The genes in the turquoise module were designated as candidate AMS-associated genes. Finally, we identified the final set of candidate AMS-associated genes as the intersection of genes derived from WGCNA, different gene expression and scRNA-seq (Figure 3B; Supplementary Table S5).

### Development, validation, and assessment of the AMS diagnostic model

**Figure 4:**
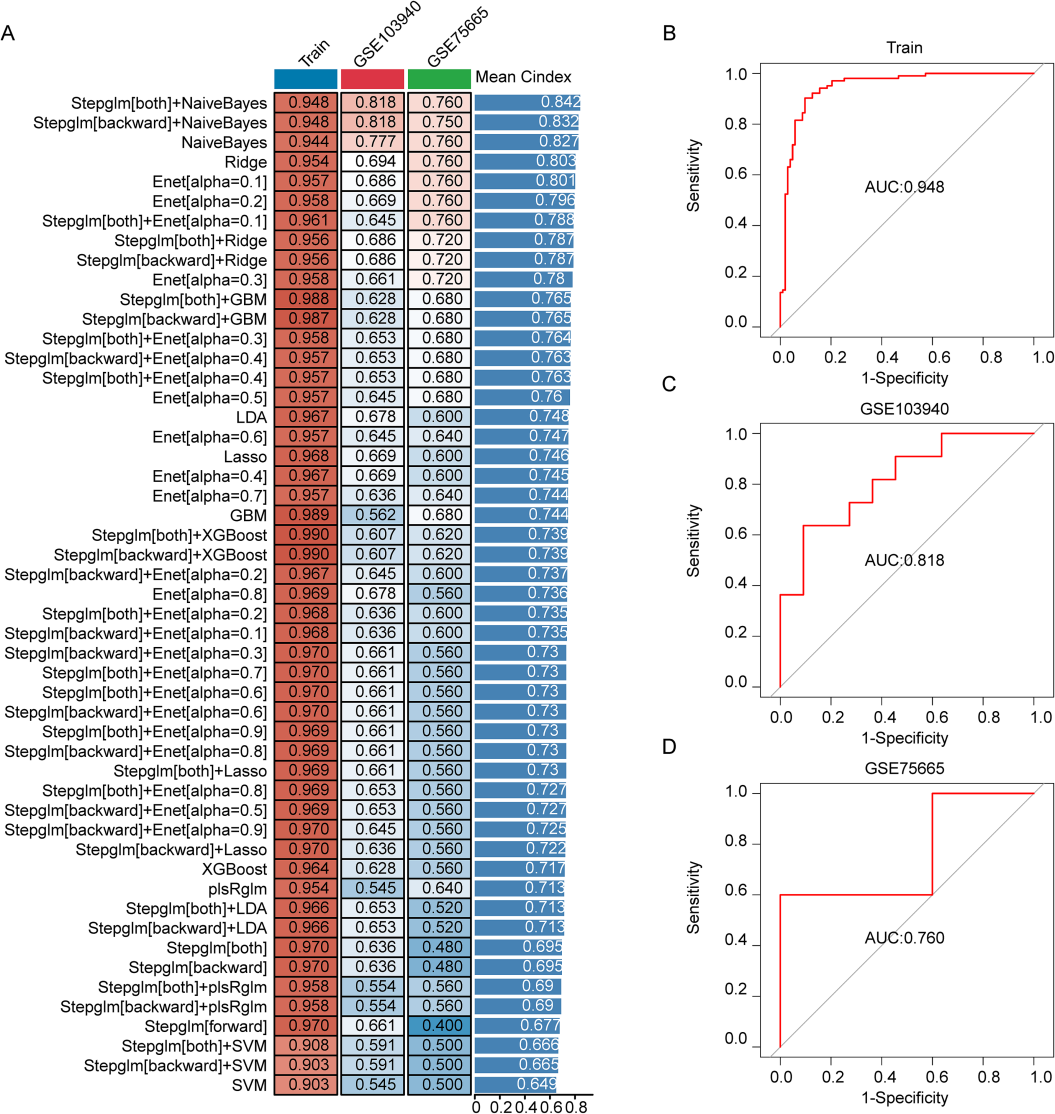
Development, validation, and assessment of the AMS Diagnostic Model. **(A)** The combination of machine learning predictive models calculated the C-index for each model on the training set (*n* = 160) and the validation sets (GSE103940 = 22; GSE75665 = 10). **(B–D)** The ROC curves showed the prediction accuracy of the diagnostic model in the training cohort **(B)**, GSE103940 cohort **(C)**, and GSE75665 cohort **(D)**.

The 12 AMS related genes were screened via both scRNA-seq and bulk RNA-seq analyses served as input features for 113 distinct machine learning model combinations to construct a diagnostic model for AMS. The models were trained on a cohort comprising 80 AMS patients and 80 controls. For external validation, datasets GSE103940 (10 AMS cases and 10 healthy controls) and GSE75665 (5 AMS cases and 5 healthy controls) were used. To assess performance, model efficacy was quantified via the concordance index (C-index) and the area under the curve (AUC) value (Figure 4A). Among the 113 combinations, the Stepglm[both] + NaiveBayes algorithm achieved the highest mean C-index of 0.842 and an AUC of 0.948 in the training cohort (Figure 4B). Furthermore, during external validation, this algorithm maintained robust performance, with AUC values of 0.818 (for GSE103940) and 0.760 (for GSE75665), respectively (Figures 4C,D). The Brier scores for both training and validation sets were below 0.25. Moreover, the Hosmer-Lemeshow test *p* value was greater than 0.05 (Supplementary Table S6). Together, these results suggested excellent model calibration. Model performance was further evaluated using the confusion matrix (Supplementary Figure S1) and standard metrics including accuracy, precision, recall, and F1-score (Supplementary Table S7). Accuracy reached 90% (training set), 81.8% (GSE103940), and 80% (GSE75665), with all datasets achieving ≥80%. Similarly, recall (88.1, 88.9, 80.0%) and F1-scores (all ≥80%) surpassed the 80% threshold consistently. These results demonstrate low false negative rates and support the model’s utility in early disease screening. The final Stepglm[both] + NaiveBayes model incorporated eight biomarker genes: ATP6V0C, BCL2A1, CD52, CSTA, GZMA, HINT1, PFDN5, RNF11. Additionally, we confirmed the expression of these genes by qPCR (Supplementary Figure S2).

### Exploring the potential mechanism of AMS associated signatures

**Figure 5:**
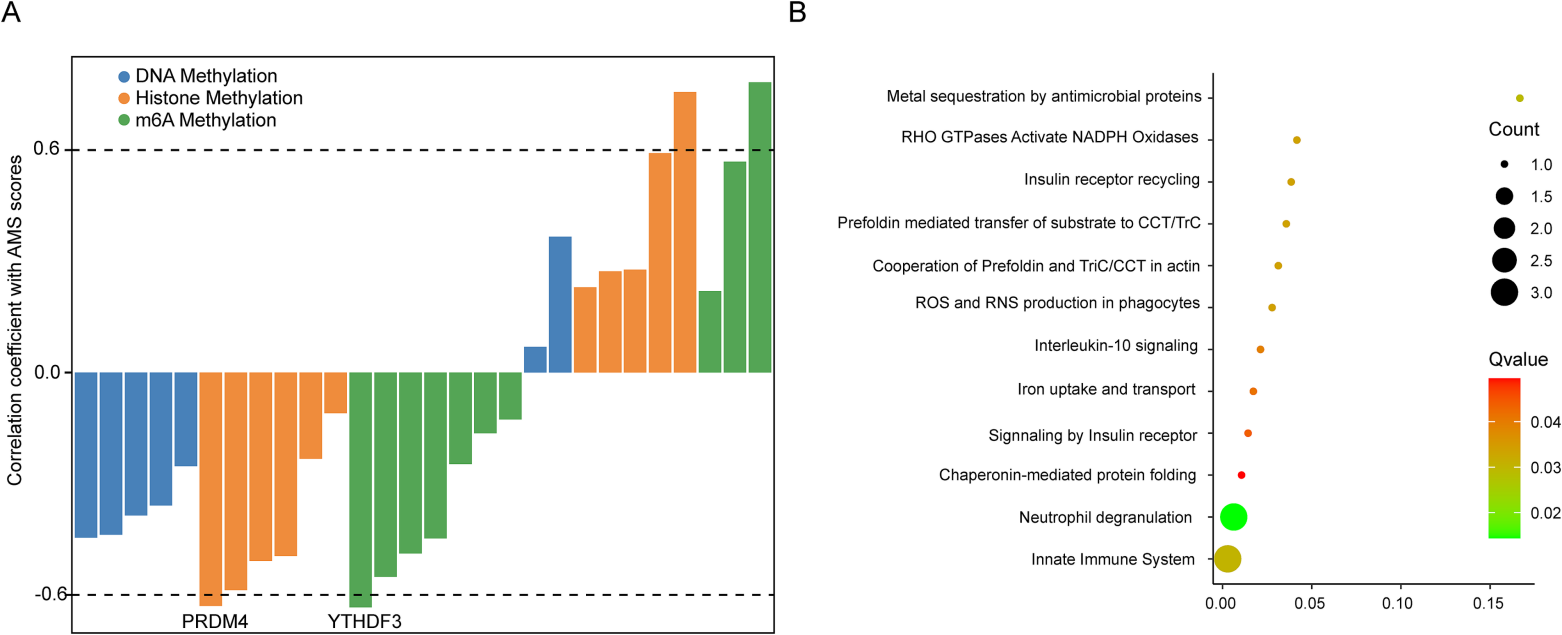
Exploring the potential mechanism of AMS associated signatures. **(A)** Spearman correlation of the expression of known epigenetic regulators with AMS scores (average expression of ATP6V0C, BCL2A1, CD52, CSTA, GZMA, HINT1, PFDN5, and RNF11) in 96 AMS samples. **(B)** The KEGG enrichment of AMS associated signatures. The significantly enrichment pathways were identified by the cutoff of adj *p* < 0.05.

To identify underlying mechanisms upstream of the AMS-associated signature, we defined an AMS score based on the average expression of eight signature genes (ATP6V0C, BCL2A1, CD52, CSTA, GZMA, HINT1, PFDN5, RNF11). This AMS score negatively correlated with the expression of two key epigenetic regulators: the histone methylation regulator PRDM4 and the m6A methylation regulator YTHDF3 (Figure 5A). These results indicated that AMS progression might be epigenetically regulated. Furthermore, pathway analysis revealed significant enrichment of these signature genes in immune-related signaling pathways and oxidative stress (adjusted *p* < 0.05) (Figure 5B).

## Discussion

AMS is the most common disease encountered at high altitudes, which typically occurs shortly after a rapid ascent to a hypoxic environment. However, the diagnosis of AMS mainly depends on a self-questionnaire, revealing the need for reliable biomarkers for AMS (23). Therefore, early, rapid, and accurate diagnosis of AMS is essential to effectively alleviate symptoms and prevent disease progression. In this study, we established a robust diagnostic model through a computational framework integrating single-cell RNA sequencing (scRNA-seq) and bulk RNA-seq data via machine learning methodologies.

To identify genes associated with AMS, we employed an integrated approach utilizing both bulk RNA-seq and single-cell RNA-seq (scRNA-seq) data. Our initial characterization of the AMS immune microenvironment revealed elevated levels of both myeloid-derived cells (MD) and platelet (PLT) activity compared to normal controls. This finding aligns with recent peripheral blood scRNA-seq studies of AMS (24). Myeloid cells, primarily neutrophils and monocytes/macrophages, played a crucial role in immune defense and coagulation homeostasis (25, 26). In the progress of AMS, these cells became activated and mediate associated inflammatory responses (27, 28). Similarly, platelets undergo significant activation upon rapid ascent to high altitude, characterized by elevated levels of the activation markers CD62P (P-selectin) and TXB_2_ (thromboxane B_2_) (29, 30). This activation promotes microthrombus formation and vasoconstriction, thereby exacerbating AMS symptoms like headache and pulmonary edema. Importantly, platelet activation levels have been shown to be markedly higher in patients with high-altitude pulmonary edema (31, 32). Given the central role of these cellular changes in AMS pathophysiology, we sought to define a robust set of AMS-associated genes from the DEGs among MD and PLT. To enhance the reliability of the candidate gene set identified from the scRNA-seq analysis, we performed additional screening using an independent bulk RNA-seq dataset. Together, we screened the AMS related genes for establishing diagnostic by scRNA-seq and bulk RNA-seq data.

We constructed a machine learning model to predict AMS using transcriptomic data. After systematically quantifying 113 combinations of machine learning algorithms (33, 34), we identified the Stepglm[both] + NaiveBayes diagnostic model as the best performer. This model achieved outstanding accuracy, with an AUC of 0.948 in the training cohort, and maintained clinical validity in external validation (AUC = 0.818 and 0.760). Moreover, calibration measures demonstrated robustness, featuring Brier scores below 0.25 and a Hosmer-Lemeshow *p* > 0.05 for both training and validation sets. For model comparison, we systematically reviewed AMS diagnostic models from the past 5 years, categorizing them into six groups: clinical, physiological/biochemical, transcriptomic, metabolomic, proteomic, and combined indicators (Supplementary Table S8). While objective indicators (e.g., clinical and physiological/biochemical) have been explored, they often require specialized equipment and expert involvement, limiting practicality in high-altitude settings. Although peripheral capillary oxygen saturation (SpO₂) shows promise as an early warning parameter, significant individual variability precludes its use as a definitive diagnostic criterion (35). Transcriptomics offers distinct advantages in plateau research, supported by the mature application of portable sequencers in field studies (36–38) and the demonstrated efficacy of our machine learning framework for early disease diagnosis. However, to enhance widespread adoption and ensure reliability, further large-scale validation across multi-center settings is essential.

The diagnostic model identified eight key genes (ATP6V0C, BCL2A1, CD52, CSTA, GZMA, HINT1, PFDN5, RNF11) implicated in immune homeostasis, extracellular matrix (ECM) remodeling, and signal transduction. Their expression profiles accurately reflect pathophysiological alterations induced by high-altitude hypoxia. First, the hypoxic environment disrupts immune homeostasis via a synergistic network involving BCL2A1, CD52, and GZMA. BCL2A1 suppresses mitochondrial apoptosis, prolonging neutrophil and monocyte survival and amplifying inflammation (39). CD52 regulates T-cell activation and migration, while GZMA mediates cytotoxic responses against damaged cells (40, 41). Together, they sustain pathological immune responses, potentially relevant to interventions such as transfusion therapy. Second, severe acute mountain sickness (AMS) involves vascular basement membrane degradation and endothelial barrier dysfunction, primarily mediated by ATP6V0C and CSTA through ECM remodeling and protease cascades (42). ATP6V0C also maintains intracellular pH and enhances red blood cell deformability under hypoxia – a mechanism related to recombinant human erythropoietin (rHuEpo) treatment for AMS (43). Finally, cellular adaptation to hypoxia relies on hypoxia-inducible factor (HIF)-mediated transcriptional reprogramming, coordinated by HINT1, PFDN5, and RNF11. Specifically, HINT1 attenuates activator protein 1 (AP-1) activation and inhibits HIF-1α-induced transcription (44); PFDN5 stabilizes HIF structural integrity (45); and RNF11 modulates HIF-1α ubiquitination and degradation (46). Additionally, our findings demonstrated that m6A methylation regulated model genes, aligning with previous studies (47–49). This epigenetic mechanism is crucial for human adaptation to high-altitude environments and the pathogenesis of plateau-related diseases.

While our study demonstrated promising findings, two limitations warrant consideration. First, mechanistic studies using experiments were warranted to clarify the biological foundations of the eight-gene diagnostic signature in AMS pathogenesis. Second, High-altitude medical studies frequently encounter challenges in participant recruitment and stringent ethical requirements, resulting in a relatively small validation cohort sample size in the present study. Despite these limitations, this study established a conceptual framework for AMS diagnosis and offers significant implications for developing personalized treatment approaches.

## Conclusion

We developed a machine learning-based diagnostic model for AMS by integrating scRNA-seq and bulk RNA-seq data. This model advanced strategies to improve the diagnosis and management of AMS patients.

## Data Availability

The data has been uploaded to the China National Center for Bioinformation with the accession number PRJCA042779 or the figshare (https://figshare.com/s/e455f8e23afbc470432e) accession number NMDCX0002155.
