# Supplementary material for: Machine learning integration identifying an eight-gene diagnostic signature for acute mountain sickness
Source: Front Med (Lausanne). 2025 Nov 18;12:1688025. doi: 10.3389/fmed.2025.1688025 (PMC12669163; doi:10.3389/fmed.2025.1688025)
Supplement: Supplementary file 1 [file Data_Sheet_1.zip › SP20251011.docx]

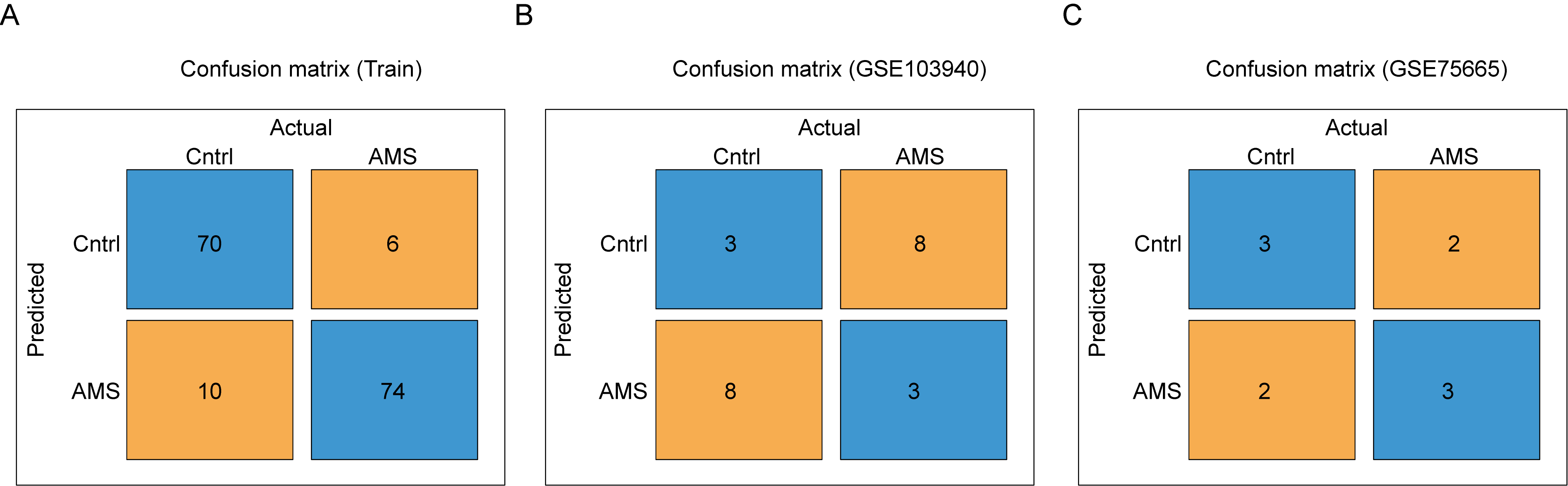


**Figure S1** Confusion matrices of the training cohort (A) and validation cohorts GSE103940 (B) and GSE75665 (C).


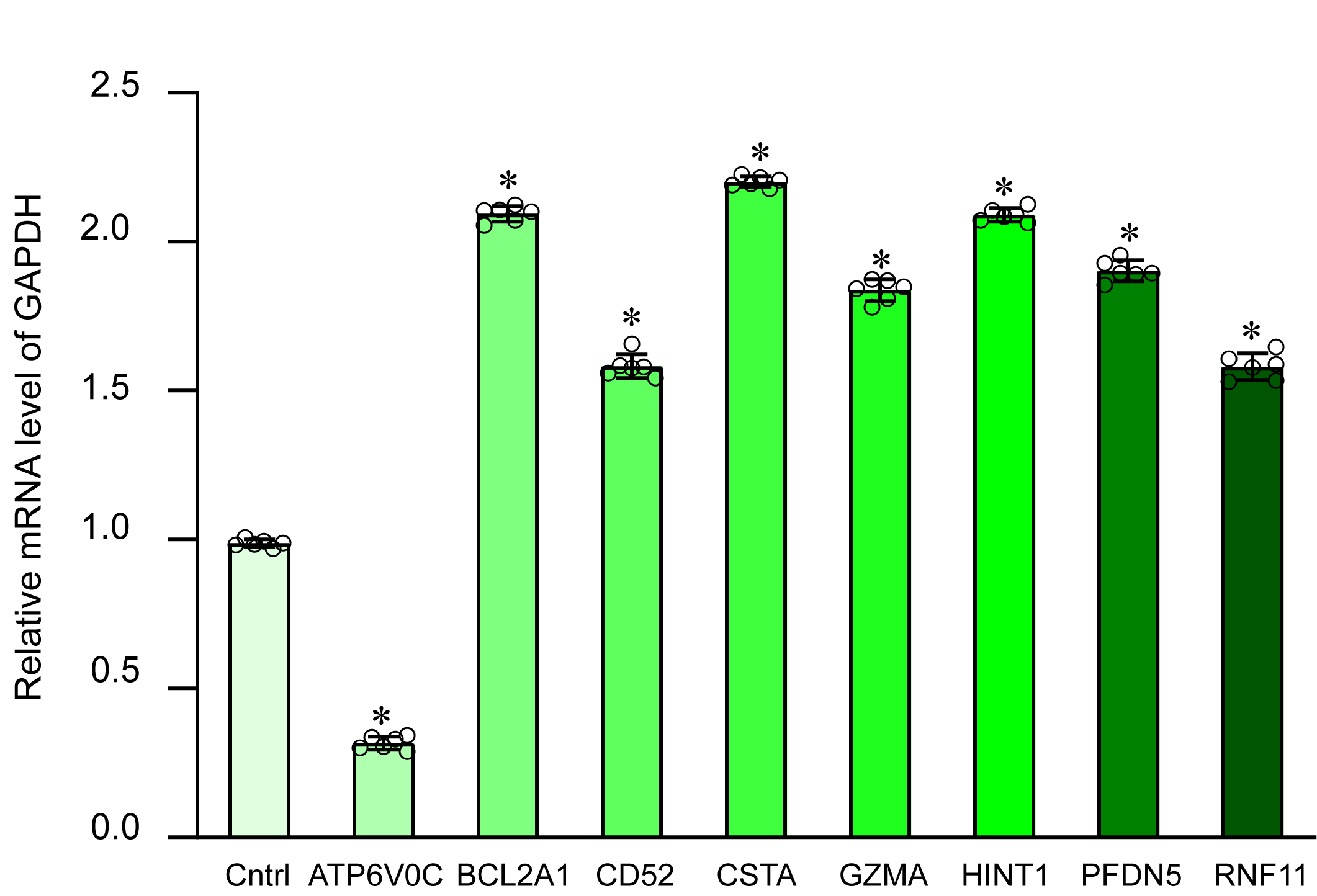


**Figure S2** Validation of HAPH-related signatures by qPCR. The results are

presented as mean ± SD (n = 6) and were normalized to the control (*P < 0.05).
